# Dynamics of social contagions with local trend imitation

**DOI:** 10.1038/s41598-018-25006-6

**Published:** 2018-05-09

**Authors:** Xuzhen Zhu, Wei Wang, Shimin Cai, H. Eugene Stanley

**Affiliations:** 1 State Key Laboratory of Networking and Switching Technology, Beijing University of Posts and Telecommunications, Beijing, 100876 China; 2 Cybersecurity Research Institute, Sichuan University, Chengdu, 610065 China; 3 Web Sciences Center, University of Electronic Science and Technology of China, Chengdu, 610054 China; 4 Big Data Research Center, University of Electronic Science and Technology of China, Chengdu, 610054 China; 5 Center for Polymer Studies and Department of Physics, Boston University, Boston, Massachusetts, 02215 USA

## Abstract

Research on social contagion dynamics has not yet included a theoretical analysis of the ubiquitous local trend imitation (LTI) characteristic. We propose a social contagion model with a tent-like adoption probability to investigate the effect of this LTI characteristic on behavior spreading. We also propose a generalized edge-based compartmental theory to describe the proposed model. Through extensive numerical simulations and theoretical analyses, we find a crossover in the phase transition: when the LTI capacity is strong, the growth of the final adoption size exhibits a second-order phase transition. When the LTI capacity is weak, we see a first-order phase transition. For a given behavioral information transmission probability, there is an optimal LTI capacity that maximizes the final adoption size. Finally we find that the above phenomena are not qualitatively affected by the heterogeneous degree distribution. Our suggested theoretical predictions agree with the simulation results.

## Introduction

The study of social contagion has attracted wide attention among researchers in the field of network science^1,2^. Studies of social contagion have focused on such subjects as behavior spreading^3^, information spreading^4^, and the contagion of sentiment^5^, and they have been both theoretical and experimental in their exploration of the essential nature of social contagion^5,6^. Unlike biological contagions (e.g., epidemic spreading)^7–9^, social contagions have a reinforcement effect^10^.

An early approach to studying social contagions is the threshold model^11,12^ based on a Markovian process without memory. Here the behavior is adopted when the fraction of neighbors who have already adopted the behavior equals or exceeds an adoption threshold. Percolation theory can be used to estimate this fraction when the initial seed size is vanishingly small^12^. When the adoption threshold is fixed, a change in mean degree size induces a saddle-node bifurcation^13,14^, and increasing the mean degree size causes the continuous growth pattern of the final adoption size to become discontinuous. Previous research has found that in the threshold model such factors as initial seed size^13^, clustering coefficient^15^, community structure^16,17^, multiplexity^18^, and network temporality^19^ all influence the social contagion process.

In real-world social contagions, memory affects behavior adoption and reinforcement. This includes both the full^3^ and partial^20^ memory of the cumulative behavioral information received from neighbors. The memory effect causes social contagions to be non-Markovian, and thus many non-Markovian social contagion models have been used to depict the social reinforcement effect when memory is included^20–25^. Recent research has found that social reinforcement originates in the memory of non-redundant information transmission^22–24^, that the growth of the final adoption size is dependent on the behavioral information transmission probability, and that it changes from continuous to discontinuous when the dynamic or structural parameters are altered.

In real-world cases, the probability that an individual will adopt a new behavior may be either positively or negatively correlated with the number of neighbors who have already adopted the behavior. For example, some style-conscious people who imitate the behavior of celebrities and adopt the latest fashions may also strive to avoid anything that has become overly-popular and ubiquitous (Leibenstein calls this the “snob effect^26^)”. Another example is when an individual habitually patronizes a restaurant with good food and a convivial atmosphere, but then avoids it when it becomes overly-popular and crowded. Both of these examples exhibit the local trend imitation (LTI) phenomenon^27–29^, i.e., the adoption probability first increases with an increase in the number of adopted neighbors and then decreases. Dodds *et al*. used a binary state model and found that the LTI characteristic causes chaos in Markovian social contagions^29^.

Because the LTI characteristic in non-Markovian social contagions has not been systematically analyzed, we here propose a social contagion model that uses the LTI characteristic to describe the dynamics of behavior spreading. The LTI characteristic is described using a tent-like adoption probability. We develop a generalized edge-based compartmental theory for quantitative validation. Both the numerical simulations and theoretical results show that the LTI characteristic strongly affects the final adoption size. In particular, when the LTI capacity is strong the system undergoes a discontinuous first-order phase transition. When it is weak the system undergoes a continuous second-order phase transition. For each spreading probability there is an optimal LTI capacity that maximizes the final adoption size. We also find that the heterogeneity level of the degree distribution does not qualitatively affect the outcome.

## Results

### Model Description

**Figure 1:**
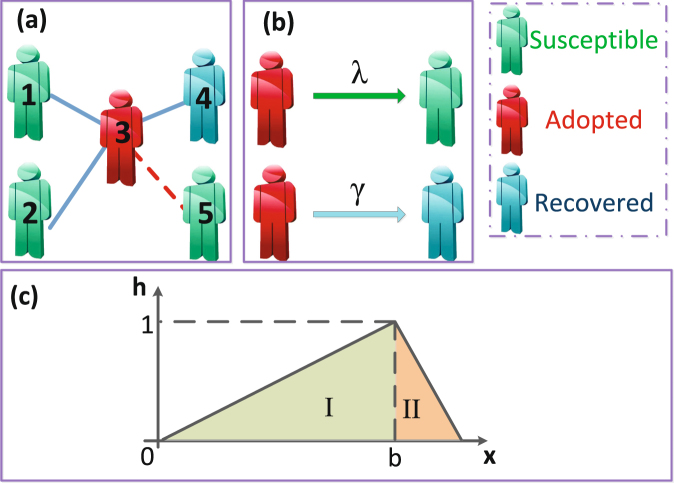
(**a**) Illustration of social contagions on complex networks. (**b**) Schematic of information transmission from the adopted individual to his each susceptible neighbor with probability *λ* and state change from being adopted to recovered with probability *γ*. (**c**) Tent-like behavior adoption probability. Notations *b* and *x* indicate local trend imitation capacity and the ratio of adopted informants, respectively. In region I, the adoption probability increases with *x*. In region II, the adoption probability decreases with *x*.

To include the LTI characteristic in social contagions, we use a tent-like function *h*(*x*, *b*) as the behavior adoption probability,

1 $${h}({x},{b})=\{\begin{array}{cc}\frac{x}{b}, & 0 < x\le b,\\ \frac{1-x}{1-b}, & b < x < 1,\end{array}$$

where *x* is the ratio between an individual’s received information and their degree. The parameter *b* is the LTI capacity of an individual. When 0 < *x* ≤ *b*, i.e., region I in Fig. 1(c), the adoption probability increases with *x*. Thus region I is the *promotion region*. When *b* < *x* < 1, i.e., region II in Fig. 1(c), the adoption probability decreases with x. Region II is the *depression regio*n. Small *b* increases the LTI capacity, and when the value of *b* is large, the LTI capacity decreases^29^.

We here use a generalized susceptible-adopted-recovered (SAR) model^22–24^ to describe behavior spreading in complex networks with *N* nodes and a degree distribution *P*(*k*). Figure 1(a) shows that at any given time each individual is in either a susceptible (S), adopted (A), or recovered (R) state. An individual in the susceptible state has not adopted the behavior. An individual in the adopted state adopts the behavior and transmits it to susceptible neighbors. An individual in the recovered state abandons the behavior and no longer transmits it.

We begin by randomly selecting an adopter seed. All other individuals are susceptible. At time step *t*, with a probability *λ* every adopted individual transmits behavioral information to every susceptible neighbor [see Fig. 1(b)]. If a susceptible neighbor receives the information, the cumulative units of information *m* collected by the neighbor increases by one, i.e., *m* → *m* + 1. When the information is transmitted through an edge, the information is not allowe to retransmit along the same edge, i.e., because repetitive transmission of the same information along the same edge does not enhance the legitimacy of the behavior, only non-redundant information transmission is permitted^3^. The probability that the susceptible individual will adopt the behavior is *h*(*m*/*k*, *b*), where *k* is the degree of the susceptible individual. The behavior spreading process is non-Markovian because every susceptible node remembers the cumulative units of information. At the same time step, adopted individuals no longer transmit the information and enter the recovered state with a probability *γ* [see Fig. 1(b)].

### Theoretical analysis

Our proposed model is inspired by refs^22,30,31^. We develop a generalized edge-based compartmental theory and define mathematical symbols *S*(*t*), *A*(*t*), and *R*(*t*) to be the fraction of individuals in the susceptible, adopted, and recovered states at time step *t*, respectively.

For convenience, we denote *u* a randomly chosen individual and *v* a randomly chosen neighbor of *u*. We assume that when *u* is in the cavity state^32^ it receives behavioral information from adopted neighbors but does not transmit it. We define *θ*(*t*) to be the probability that a randomly chosen neighbor *v* of *u* by time *t* has not transmitted the behavioral information to *u* along a randomly selected edge. By time *t*, the individual *u* with degree *k* has received *m* units of behavioral information from neighbors with a probability

2 $${\varphi }_{m}(k,t)=(\begin{array}{c}k\\ m\end{array}){[\theta (t)]}^{k-m}{\mathrm{[1}-\theta (t)]}^{m}\mathrm{.}$$

Individual *u* with a degree *k* and *m* units of received information remains susceptible with a probability $\prod _{j\mathrm{=0}}^{m}\mathrm{[1}-h(\frac{j}{k},b)]$. The probability that an individual *u* with degree *k* has received *m* units of information and by time *t* is still in in susceptible state is

3 $$\begin{array}{ll}S(k,t) & =\sum _{m\mathrm{=0}}^{k}{\varphi }_{m}(k,t)\prod _{j\mathrm{=0}}^{m}[1-h(\frac{j}{k},b)]\\  & =\sum _{m\mathrm{=0}}^{\lfloor bk\rfloor }{\varphi }_{m}(k,t)\prod _{j\mathrm{=0}}^{m}[1-\frac{j}{bk}]\\  & +\sum _{m=\lceil bk\rceil }^{k}{\varphi }_{m}(k,t)\prod _{j\mathrm{=0}}^{\lceil bk\rceil }[1-\frac{j}{bk}]\prod _{j=\lceil bk\rceil }^{m}[1-\frac{1-\frac{j}{k}}{1-b}]\mathrm{.}\end{array}$$

Taking into consideration all possible degrees *k*, we calculate the total ratio of susceptible individuals to be

4 $$S(t)=\sum _{k}P(k)S(k,t\mathrm{).}$$

A randomly chosen neighbor *v* of individual *u* is either susceptible, adopted, or recovered, and thus *θ*(*t*) can be divided, i.e.,

5 $$\theta (t)={\xi }_{S}(t)+{\xi }_{A}(t)+{\xi }_{R}(t),$$

where *ξ*_*S*_(*t*), *ξ*_*A*_(*t*), and *ξ*_*R*_(*t*) denote the probabilities that a neighbor of individual *u* in the cavity state is susceptible, adopted, or recovered, respectively, and thus has not transmitted the information to individual *u* through an edge by time *t*.

When *v* with degree *k*′ is initially susceptible, it cannot transmit behavioral information to *u*, but can receive information from all *k*′ − 1 neighbors of *v* except susceptible *u*. Thus we determine the probability that neighbor *v* of individual *u* by time *t* has received *m* units of information to be

6 $${\varphi }_{m}(k^{\prime} -1,\,t)=(\frac{k^{\prime} -1}{m}){[\theta (t)]}^{k^{\prime} -m-1}{\mathrm{[1}-\theta (t)]}^{m}\mathrm{.}$$

After taking into consideration all possible values of *m*, we determine the probability that a randomly chosen neighbor *v* with degree *k*′ remains susceptible to be

7 $$\begin{array}{ll}{\rm{\Theta }}(k^{\prime} ,\,t) & =\sum _{m=0}^{k^{\prime} -1}{\varphi }_{m}(k^{\prime} -\mathrm{1,}\,t)\prod _{j=0}^{m}[1-h(\frac{j}{k^{\prime} },b)]\\  & =\sum _{m=0}^{\lfloor bk^{\prime} \rfloor }{\varphi }_{m}(k^{\prime} -\mathrm{1,}\,t)\prod _{j=0}^{m}[1-\frac{j}{bk^{\prime} }]\\  & +\sum _{m=\lceil bk^{\prime} \rceil }^{k^{\prime} -1}{\varphi }_{m}(k^{\prime} -\mathrm{1,}\,t)\prod _{j=0}^{\lceil bk^{\prime} \rceil }[1-\frac{j}{bk^{\prime} }]\prod _{j=\lceil bk^{\prime} \rceil }^{m}[1-\frac{1-\frac{j}{k^{\prime} }}{1-b}]\mathrm{.}\end{array}$$

In an uncorrelated network, an edge connects an individual of degree *k*′ with probability *k*′*P*(*k*′)/〈*k*〉, where 〈*k*〉 is the average degree. We obtain

8 $${\xi }_{S}(t)=\sum _{k^{\prime} }\frac{k^{\prime} P(k^{\prime} )}{\langle k\rangle }{\rm{\Theta }}(k^{\prime} ,t\mathrm{).}$$

If an adopted individual transmits behavioral information through an edge with probability *λ*, *θ*(*t*) does not fulfill the definition, and the decrease of the fraction of *θ*(*t*) equals *λξ*_*A*_(*t*), which is

9 $$\frac{d\theta (t)}{dt}=-\,\lambda {\xi }_{A}(t\mathrm{).}$$

If an adopted individual does not transmit the behavioral information through any edge with probability 1 − *λ* but moves into the recovered state with probability *γ*, *ξ*_*R*_(*t*) will consequently increase. We thus obtain

10 $$\frac{d{\xi }_{R}(t)}{dt}=\gamma \mathrm{(1}-\lambda ){\xi }_{A}(t\mathrm{).}$$

Using Eqs (9) and (10), and the initial conditions of *θ*(0) = 1 and *ξ*_*R*_(0) = 0, we obtain

11 $${\xi }_{R}(t)=\frac{\gamma \mathrm{[1}-\theta (t\mathrm{)](1}-\lambda )}{\lambda }\mathrm{.}$$

Substituting *ξ*_*S*_(*t*), *ξ*_*A*_(*t*) and *ξ*_*R*_(*t*) of Eq. (5) into Eqs (8, 9 and 11), respectively, we find the time evolution of *θ*(*t*) to be

12 $$\frac{d\theta (t)}{dt}=-\,\lambda [\theta (t)-\sum _{k^{\prime} }\frac{k^{\prime} P(k^{\prime} )}{\langle k\rangle }{\rm{\Theta }}(k^{\prime} ,t)]+\gamma \mathrm{[1}-\theta (t\mathrm{)](1}-\lambda \mathrm{).}$$

At each time step *t*, some susceptible individuals adopt the behavior and some adopted individuals move into the recovered state. Note that the growth of *A*(*t*) is equivalent to the decrease of *S*(*t*) minus the fraction of adopted individuals that with probability *γ* enter into the recovered state. Thus the time evolution of *A*(*t*) is

13 $$\begin{array}{ll}\frac{dA(t)}{dt} & =-\frac{dS(t)}{dt}-\gamma A(t)\\  & =-\sum _{k}P(k)\frac{dS(k,t)}{dt}-\gamma A(t),\end{array}$$

where

14 $$\begin{array}{l}\frac{dS(k,t)}{dt}=\sum _{m=0}^{\lfloor bk\rfloor }{\rm{\Psi }}(t)\prod _{j=0}^{m}\mathrm{[1}-\frac{j}{bk}]+\sum _{m=\lceil bk\rceil }^{k}{\rm{\Psi }}(t)\prod _{j=0}^{\lceil bk\rceil }\mathrm{[1}-\frac{j}{bk}]\prod _{j=\lceil bk\rceil }^{m}[1-\frac{1-\frac{j}{k}}{1-b}]\end{array}$$

and

15 $$\begin{array}{ll}{\rm{\Psi }}(t) & =\,\frac{d{\varphi }_{m}(k,t)}{dt}\\  & =\,(\begin{array}{c}k\\ m\end{array})\{(k-m)[\theta (t{)]}^{k-m-1}{\mathrm{[1}-\theta (t)]}^{m}-m{[\theta (t)]}^{k-m}{\mathrm{[1}-\theta (t)]}^{m-1}\}\mathrm{.}\end{array}$$

The time evolution of *R*(*t*) is

16 $$\frac{dR(t)}{dt}=\gamma A(t\mathrm{).}$$

Equations (2–4) and (12–13) describe social contagion in terms of LTI, and they can be used to compute the fraction of each state at any arbitrary time step. When *t* → ∞, we find the final adoption size *R*(∞).

In the final state, we find that

17 $$\theta (\infty )=\sum _{k^{\prime} }\frac{k^{\prime} P(k^{\prime} )}{\langle k\rangle }{\rm{\Theta }}(k^{\prime} ,\infty )+\frac{\gamma \mathrm{[1}-\theta (\infty \mathrm{)](1}-\lambda )}{\lambda }\mathrm{.}$$

Note that *θ*(*t*) decreases with *t* when adopted individuals continually transmit the behavioral information to neighbors. Thus when there is more than one stable fixed point in Eq. (17) only the maximum stable fixed point is physically meaningful. Inserting this value into Eqs (2)–(4) gives us the steady value of the susceptible density *S*(∞) and the final adoption size *R*(∞).

**Figure 2:**
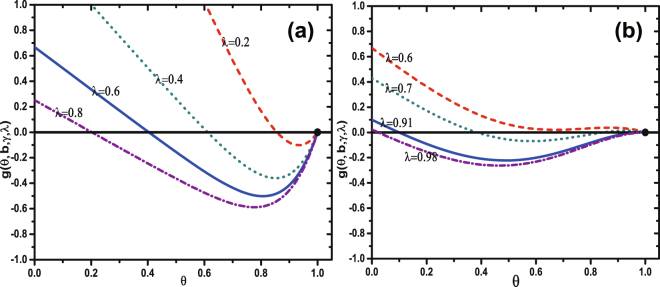
Demonstration of graphical solutions of Eq. (18) for *b* = 0.1 (**a**) and *b* = 0.9 (**b**). The horizontal axis are colored black and the tangent points are denoted as black dots.

Numerically solving Eq. (18), we find that either (i) it has only two solutions for any value of *λ* [see Fig. 2(a)], or (ii) it has either one or three solutions for different values of *λ* [see Fig. 2(b)]. When (i) occurs, the trivial solution of Eq. (17) is *θ*(∞) = 1 and there is no global behavior adoption. When global behavior occurs, Eq. (17) has a non-trivial solution *θ*(∞) < 1. At the critical point, the equation

18 $$\begin{array}{l}g[\theta (\infty ),b,\gamma ,\lambda ]=\sum _{k^{\prime} =1}^{N-1}\frac{k^{\prime} P(k^{\prime} )}{\langle k\rangle }{\rm{\Theta }}(k^{\prime} ,\infty )+\frac{\gamma \mathrm{[1}-\theta (\infty \mathrm{)](1}-\lambda )}{\lambda }-\theta (\infty )\end{array}$$

is tangent to the horizontal axis at *θ*(∞) = 1. Thus we find the critical condition of the general social contagion model to be

19 $$\frac{dg}{d\theta (\infty )}{|}_{\theta (\infty \mathrm{)=1}}=0.$$

Using Eq. (19) we find the continuous critical information transmission probability to be

20 $${\lambda }_{c}^{{\rm{II}}}=\frac{\gamma }{{\rm{\Gamma }}+\gamma -1},$$

where

$${\rm{\Gamma }}=\sum _{k^{\prime} }\frac{k^{\prime} P(k^{\prime} )}{\langle k\rangle }(k^{\prime} -\mathrm{1)}h(\frac{1}{k^{\prime} },b)\mathrm{.}$$

Numerically solving Eqs (17–20), we find ${\lambda }_{c}^{{\rm{II}}}$ to be a given adoption probability *h*(*x*, *b*). Here ${\lambda }_{c}^{{\rm{II}}}$ is associated with adoption probability *h*(*x*, *b*), recovery probability *γ*, degree distribution *P*(*k*), and average degree 〈*k*〉.

In the second scenario, Eq. (17) can have three solutions, and a saddle-node bifurcation can occur [see Fig. 2(b)], which has been found using the mean network degree^13^. Only the largest solution is valid because only that value can be achieved physically. Otherwise the fixed point is the valid solution. Changing *λ* causes the physically meaningful stable solution of *θ*(∞) jump to an alternate value. A discontinuous growth pattern of *R*(∞) with *λ* emerges, and solving Eqs (17–20) gives us the critical transmission probability ${\lambda }_{c}^{{\rm{II}}}$ at which the discontinuity occurs. When *b* = 0.9, for different values of *λ* the function *g*[*θ*(∞), *b*, *γ*, *λ*] is tangent to the horizontal axis at ${\lambda }_{c}^{{\rm{II}}}=0.91$. When $\lambda  < {\lambda }_{c}^{{\rm{II}}}$, if there are three fixed points in Eq. (17), e.g., *λ* = 0.7, the largest is the solution. When $\lambda ={\lambda }_{c}^{{\rm{II}}}$, the tangent point is the solution. When $\lambda  > {\lambda }_{c}^{{\rm{II}}}$, e.g., *λ* = 0.98, the only fixed point is the solution of Eq. (17), which abruptly drops to a small value from a large value at $\lambda ={\lambda }_{c}^{{\rm{II}}}$ and causes a discontinuous change in *R*(∞).

For a given *P*(*k*), *λ*, and *γ*, and using an analytical method similar to Eq. (20), we set *f*(*b*) = Γ to be

21 $$f(b)=\sum _{k^{\prime} }\frac{k^{\prime} P(k^{\prime} )}{\langle k\rangle }(k^{\prime} -\mathrm{1)}h(\frac{1}{k^{\prime} },b),$$

and

22 $$f(b)=\frac{\gamma +\lambda -\gamma \lambda }{\lambda }\mathrm{.}$$

Using Eqs (21) and (22) gives us the critical *b* solution

23 $${b}_{c}^{{\rm{II}}}={f}^{-1}(\frac{\gamma +\lambda -\gamma \lambda }{\lambda })\mathrm{.}$$

From this theoretical analysis and using non-redundant memory, the social contagion with an LTI character displays first and second-order phase transitions.

### Numerical simulations

Extensive experiments have been performed on ER and SF, where the network size, mean degree, and recovered probability are *N* = 10^4^, 〈*k*〉 = 10, and *γ* = 1.0, respectively.

**Figure 3:**
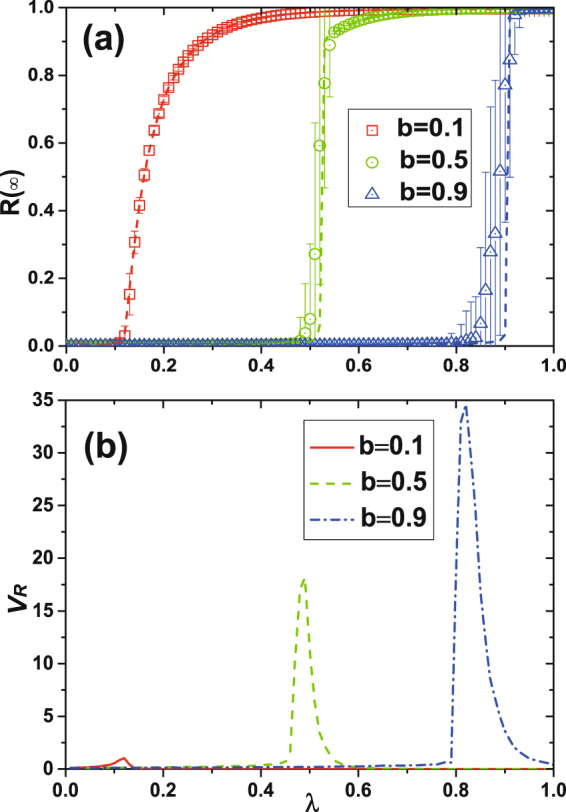
Illustration of effects of dynamical parameter *b* with arbitrary *λ*, where *b* is the LTI capacity parameter. (**a**) Under different *b*, the increase manner of final adoption size changes from continuity at small *b* (e.g. *b* = 0.1) to discontinuity at large *b* (e.g. *b* = 0.5), which embody the second-order and first-order phase transition. (**b**) *v*_*R*_ numerically exhibits the fluctuation of *R*(∞) to intuitively emphasize the critical ${\lambda }_{c}^{{\rm{II}}}$ corresponding to the peak. The higher peak, the more abrupt the discontinuity of *R*(∞) is (see *b* = 0.1, 0.5 and 0.9).

To study social contagions on ER networks, we examine the final adoption size *R*(∞) as a function of the transmission probability *λ* for different values of the LTI capacity *b* when *γ* = 1.0. Figure 3(a) shows that a bifurcation analysis of Eq. (17) reveals that the LTI capacity affects the type of phase transition. When the LTI capacity is strong, e.g., *b* = 0.1, the system exhibits a second-order phase transition, because a small *b* value indicates that a low ratio of informants can cause massive behavior adoptions even when the transmission rate *λ* is low. When the LTI capacity is weak, e.g., *b* = 0.5 or 0.9, the system exhibits a first-order phase transition, because a high *b* value indicates that a high ratio of informants and low transmission rate *λ* with a low probability of transmitting information does not substantially increase the informant ratio of susceptible individuals. When *b* is high, the transmission rate *λ* exceeds a critical point and there are massive information receptions by many individuals. The informant ratio of susceptible individuals increases rapidly, and there is an abrupt increase in behavior adoption.

We calculate the theoretical value of ${\lambda }_{c}^{{\rm{II}}}$ using Eqs (17–19). To locate the numerical critical points, we examine the relative variance *v*_*R*_ of *R*(∞) shown in Fig. 3(b). The definition of *v*_*R*_ is in the Method section. Our theoretical results agree with simulation results, except when *λ* is close to the critical information transmission probability. The deviations between our predictions and the simulations are caused by network finite-size effects and strong dynamical correlations among the states of neighbors.

**Figure 4:**
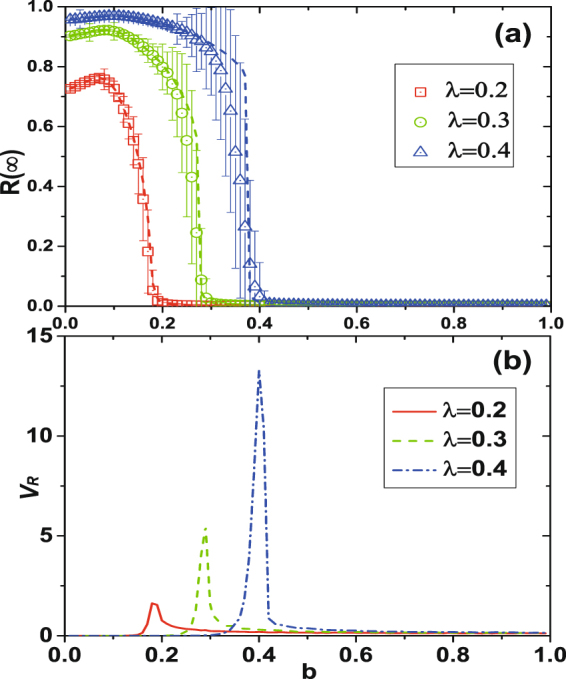
Illustration of effects of dynamical parameter *λ* with arbitrary *b*, where *λ* is behavior transmission probability and *b* is LTI capacity parameter. (**a**) Under different *λ*, the change pattern of *R*(∞) with *b* shows a first increase and then a decrease. Ultimately, the *R*(∞) vanishes to zero. (**b**) To find the critical point where the *R*(∞) vanishes, *v*_*R*_ is introduced here. Besides, the peaks of *v*_*R*_ lines correspond to critical points, and the higher the peaks the sharper the jump to zero.

Figure 4 shows an analysis of *R*(∞) versus *b* for different *λ* values. For a given *λ*, *R*(∞) changes nonmonotonically with *b*. In particular, *R*(∞) first increases with *b* and then decreases discontinuously to zero. Thus there is an optimal *b*_*o*_ value at which *R*(∞) reaches its maximum value. Taking *λ* into account, when *b* is smaller than optimal *b* the LTI capacity is strong, many susceptible neighbors become adopted, and *R*(∞) steadily increases. When *b* increases the LTI capacity decreases and is less able to inform neighbors, but *R*(∞) continues to increase until *b* exceeds optimal *b*. At optimal *b*, the informing process becomes balanced and *R*(∞) reaches a maximum. When *b* increases beyone optimal *b* the LTI capacity and informing capacity decreases, the number of informed neighbors is insufficient to support further informing, and *R*(∞) gradually declines until it reaches zero. Figure 4(b) shows that the critical point can be located by examining *v*_*R*_. Again our theoretical results agree with numerical simulation results.

**Figure 5:**
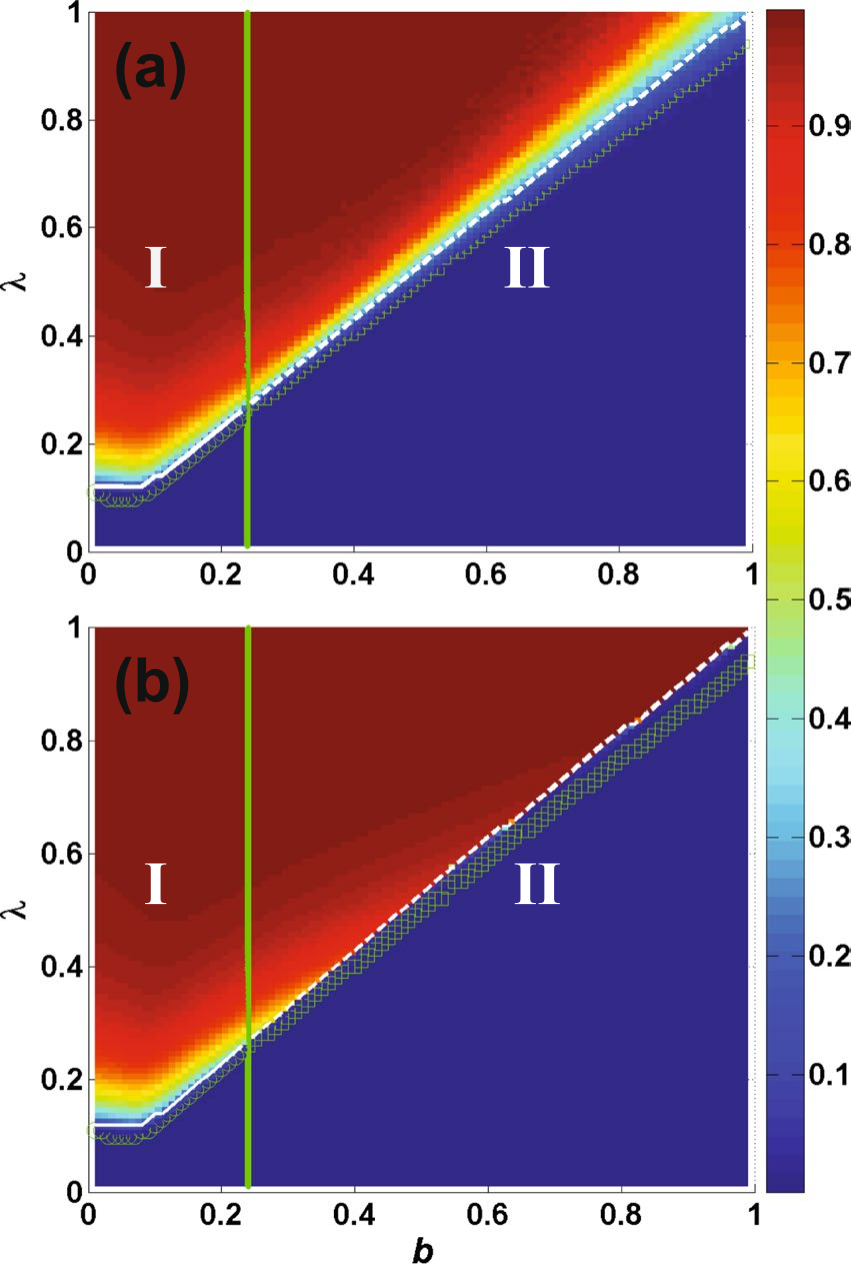
Dependence of the final adoption size *R*(∞) on *b* and *λ* on ER network. Color-coded values of *R*(∞) are obtained from numerical simulations (**a**) and theoretical solutions (**b**). Herein, the theoretical solutions are achieved through Eqs (2–4) and (9–16). The parameter plain is divided into two regions by b^*^ which is obtained from Eqs (19), (20) and (23). In regions *I*, *R*(∞) shows a continuous increase and undergoes a second-order phase transition. In contrast, *R*(∞) exhibits a discontinuous increase and undergoes a first-order phase transition in region *II*. The solid white line from theoretical method and green circles from numerical simulation all represent the critical ${\lambda }_{c}^{{\rm{II}}}$ in region *I*. And the dashed white line from theoretical method and green rectangles from numerical simulation as well denote the the critical ${\lambda }_{c}^{{\rm{II}}}$ in region *II*.

Figure 5 shows *R*(∞) on the phase transition plane (*λ*, *b*). According to the type of phase transition, the parameter plane (*λ*, *b*) is divided into two regions by the critical value of *b* (b^*^ = 0.237), which can be obtained using Eqs (18) and (20). In region I, i.e., (*b* ≤ b^*^), *R*(∞) increases continuously and exhibits a second-order phase transition. In region II, i.e., (*b* > b^*^), *R*(∞) increases discontinuously with *λ* and exhibits a first-order phase transition. There is also a crossover in the phase transition. The numerical simulation agrees with the theoretical solution.

**Figure 6:**
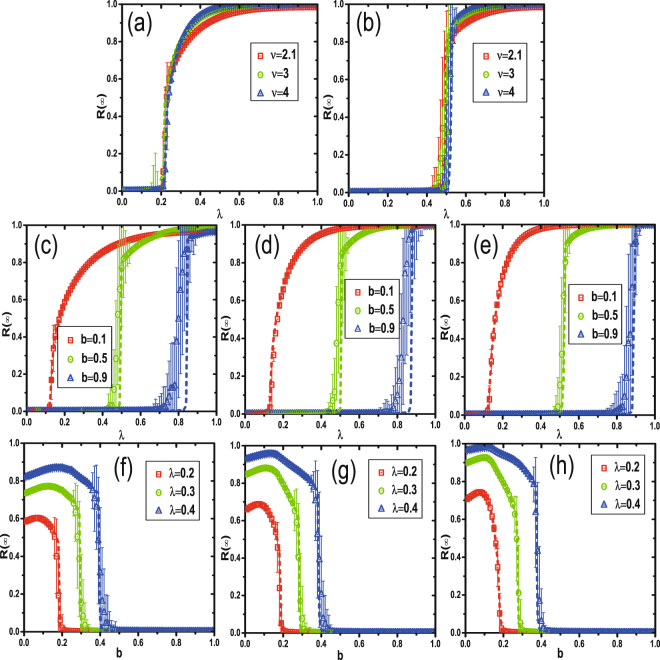
Effect of network heterogeneity in social contagion dynamics. For scale-free network with mean degree 〈*k*〉 = 10 and network size *N* = 10000, dependence of *R*(∞) on *b*, *v*, and *λ* is explored under parameter values. Subgraph (**a**) and (**b**) demonstrate the impact on *R*(∞) of separate degree exponent *v*, respectively under *b* = 0.2 and 0.5. Then, subgraph (**c**–**e**) exhibit the results of *b* influencing *R*(∞), referring to degree exponent *v* = 2.1, 3, and 4. Furthermore, for rigorousness, subgraph (**f**–**h**) proceed to show the changes of *R*(∞) based on different transmission probability *λ*, also separately under *v* = 2.1, 3, and 4. As expected, the theoretical solutions, denoted by dash line, perfectly coincide with numerical values, marked by symbols.

Figure 6 shows a study of the effects of the heterogeneity of degree distribution on social contagion. Here we focus on the SF network with different degree exponents *v*. We set the average degree and network size to be 〈*k*〉 = 10 and *N* = 10^4^, respectively. Figure 6(a,b) show that the heterogeneity of degree distribution does not change the type of phase transition when *b* = 0.2 and 0.5, respectively.

Figure 6(a) shows that when *b* = 0.2 the increase of *R*(∞) exhibits a change from a discontinuous first-order to a continuous second-order phase transition, when *v* rises from 2.1 to 4. Figure 6(b) shows, in contrast, when *b* = 0.5, *R*(∞) increases and exhibits the same pattern of first-order phase transition at any *v* value and jumps higher at the critical ${\lambda }_{c}^{{\rm{II}}}$ when *v* = 2.1, 3, and 4. Figure 6(c–e) show that when *v* = 2.1, *v* = 3, and *v* = 4 increasing *b* also changes the growth pattern of *R*(∞) from a second-order phase transition to a first-order, but that the final adoption size increases with *v*, i.e., the heterogeneous degree distribution does not impede the change in phase transition. Figure 6(f–h) show that when *v* = 2.1, *v* = 3, and *v* = 4 the transmission probability *λ* influences the final range of *R*(∞) that increases the number of individuals when *v* is higher. For all three values and under each *λ* the optimal LTI capacity parameter *b* that maximizes *R*(∞) appears, and the critical LTI capacity point of ${b}_{c}^{{\rm{II}}}$ reduces *R*(∞) to zero, even though a higher value of *v* promotes a wider spreading of behavior information. A heterogeneous degree distribution always causes a change of phase transition as in (c)–(e) and of optimal and critical LTI capacity parameters as in (f)–(h). In addition, the theoretical solutions (dashed lines) agree with the numerical values (symbols) in all subsections of Fig. 6.

## Discussion

The local trend imitation (LTI) phenomenon is ubiquitous and strongly affects the dynamics of social contagions. We have proposed a social contagion model that uses a tent-like adoption function to systematically study the role of LTI. We use an edge-based compartmental theory to describe the model and find that the theoretical predictions agree with the numerical simulations. We also perform extensive numerical simulations on ER networks. We find that when the LTI capacity is weak the final adoption size grows discontinuously, i.e., the system exhibits a first-order transition, but when the LTI capacity is strong the size of the final behavior adoption grows continuously, i.e., the system exhibits a second-order phase transition. Thus there is a crossover in the phase transition type. For a given probability of information transmission, there is an optimal LTI capacity at which the final adoption size is markedly increased. We also find that degree heterogeneity does not qualitatively alter these phenomena.

## Method

The relative variance *v*_*R*_ is designed numerically to determine the size-dependent critical values ${\lambda }_{c}^{{\rm{II}}}$ and ${b}_{c}^{{\rm{II}}}$. The relative variance of *R*(∞)^33^ is defined

24 $${v}_{R}=\frac{\langle {(R(\infty )-\langle R(\infty )\rangle )}^{2}\rangle }{{\langle R(\infty )\rangle }^{2}},$$

where 〈...〉 is the ensemble average. The value of *v*_*R*_ shows the peaks (indicating phase transitions) of *R*(∞) when a dynamical parameter is varied. Thus we know that the ${\lambda }_{c}^{{\rm{II}}}$ and ${b}_{c}^{{\rm{II}}}$ correspond to the maximum *v*_*R*_ under different values of *λ* and *b*, respectively.
